# Differential susceptibility to colonic ulceration in mice with genetic deletion of *Prostaglandin E synthase*

**DOI:** 10.21203/rs.3.rs-6977322/v1

**Published:** 2025-07-07

**Authors:** Masako Nakanishi, Michael J. Martinez, Patrycja Sztachelski, Marion Leclerc, Daniel W. Rosenberg

**Affiliations:** University of Connecticut Health Center: UConn Health

**Keywords:** PGE2, mPGES-1, colonic ulceration, mouse models, inflammatory bowel disease (IBD), non-steroidal anti-inflammatory drugs (NSAIDs), gut microbiome

## Abstract

**Background:**

Prostaglandin E_2_ (PGE_2_) exerts pleiotropic and context-dependent effects on inflammation, cancer and maintenance of intestinal mucosal homeostasis. To further define its role in intestinal diseases, we genetically inactivated its rate-limiting synthesis, *Ptges*, in two mouse lines. An unexpected phenotype consisting of spontaneous mucosal ulceration was found exclusively in the colons of strain A mice. This study aims to characterize the phenotype that may have a clinical relevance to NSAID-induced enteropathies.

**Methods:**

Mucosal ulcerations were characterized in *Ptges*-deficient mice maintained on strain A (*A/D:KO*) and C57BL/6 (*B6D:KO*) backgrounds. RNA sequencing of colons identified inflammatory signatures in sensitive *A/D:KO* mice. Microbial dysbiosis was evaluated in the fecal stream using 16S rRNA sequencing. The potential role of genetic and environmental factors in the etiology of differential susceptibility to colonic ulceration was examined through the co-housing experiment and by generating F1 hybrid of *A/D:KO* and *B6D:KO* mice.

**Results:**

Progressive colonic ulcerations develop spontaneously in *A/D:KO* mice, a phenotype that is absent in *B6D:KO* mice. RNAseq analysis revealed robust expression of pro-inflammatory genes in *A/D:KO* mice prior to the development of tissue damage, suggesting a subtle defect in intrinsic immune regulation. In *B6D:KO* colons, there was potent enrichment of genes associated with protection against mucosal injury in. While distinct gut microbial community structures were identified, co-housing of these mice did not rescue the inflammatory phenotype in *A/D:KO*, nor confer sensitivity to the colons of *B6D:KO* mice. However, F1 hybrids of *A/D:KO* with *B6D:KO* mice were mostly free of colonic ulceration.

**Conclusions:**

These results suggest that genetic blockage of *Ptges* causes a dramatic shift in the inflammatory *milieu* in strain A mice, an effect that may be augmented by a complex interaction between genetic background, microbiome and metabolite imbalance. These mice may provide a genetic model for studying interindividual variability in human sensitivity to NSAID-induced colitis.

## Background

The generation of the prostanoids through the arachidonic acid (AA)-cyclooxygenase (COX) metabolic pathway regulates key aspects of cellular physiology. Among these bioactive lipids, prostaglandin E_2_ (PGE_2_) is the most abundantly produced prostanoid, impacting an array of biological processes associated with inflammation, cancer and paradoxically, cell homeostasis [1]. Blockade of COX activity by commonly used non-steroidal anti-inflammatory drugs (NSAIDs) reduces PGE_2_ levels, which has proven to be an effective strategy for managing pain and inflammation, and in some cases for preventing cancer [2, 3]. However, long-term treatment with NSAIDs can cause off-target effects, including cardiovascular toxicity and gastrointestinal (GI) ulcerations [2]. In fact, a record-based retrospective single-center study showed that approximately 50% of patients taking NSAIDs reported GI-related side effects [4].

These adverse effects are due, in part, to dysregulated synthesis of related prostanoids sharing a common metabolic precursor [5]. To specifically reduce PGE_2_ levels while sparing these other essential metabolites, we [6, 7] and others [8] have targeted the inducible terminal enzyme, microsomal PGE synthase-1 (mPGES-1; *Ptges*), as an alternative therapy for managing inflammation and cancer.

In earlier studies, we demonstrated that genetic inactivation of *Ptges* provides protection against intestinal tumor development in carcinogen-treated C57BL/6 and strain A mice, affording comparable protection to targeting COX activity with NSAIDs [6, 7, 9]. During the course of these studies, however, we unexpectedly observed spontaneous ulcerations appearing throughout the colons of strain A mice lacking *Ptges*, a phenotype that was largely absent in C57BL/6 mice with this null genotype. This observation indicates a strain-dependent differential sensitivity to *Ptges* blockade in the colon that may provide a model for interindividual patient sensitivity to long-term NSAID treatment.

Use of slow-release or enteric-coated NSAIDs (e.g., Ibuprofen, naproxen), selective Cox inhibitors (e.g., Celebrex), as well as concurrent administration of proton pump inhibitors (PPI) have reduced the incidence of upper GI complications [10]. However, they fail to protect the lower GI, particularly the colonic mucosa [11]. In fact, NSAID-associated injuries to the colonic mucosa, collectively termed *colopathy*, display erosions, ulcerations and diaphragm-like strictures, as well as perforation, obstruction and diverticulitis [12–15]. While the overall prevalence and incidence of colopathy is unclear, approximately 10% of colitis cases are related to NSAID intake [16]. Pathogenesis of NSAID-induced colopathy remains understudied, partly due to the varying risk of developing clinically significant cases within the general population [17].

To fully characterize this highly penetrant phenotype in mice and its potential translational relevance, the following study was undertaken. We report here that the localized colonic inflammation that develops in susceptible *Ptges-*deficient strain A mice closely resembles the pathology of human drug-induced colitis. Gene expression profiling of normal colonic mucosa prior to the onset of active disease has identified dysregulation of several key signaling pathways that may contribute to mucosal injury, including the activation of inflammatory networks and antibacterial responses. While fecal microbiome analysis showed marked differences in the abundance of resident microbial taxa in the parental mouse lines, co-housing of these mice did not rescue the inflammatory phenotype in strain A, nor confer sensitivity to the colons of C57BL/6 mice. However, an F1 hybrid of these mouse lines were mostly free of colonic ulceration, indicating that genetic background may be the dominant factor in the development of the colitis-like phenotype.

## Materials and Methods

### Animals.

Strain A mice (A/J) and C57BL/6J (B6) were purchased from The Jackson Laboratory (Bar Harbor, ME). *Ptges* heterozygous (HET) mice on a C57BL/6NTac background were provided by Merck Frosst Canada, Ltd. [18] and crossed to *wild-type* (*WT*) C57BL/6J for 10 generations (*B6.129P2(B6)-Ptges*^*tm1Dwr*^*/Drmn*, **B6D:HET**), and maintained as *Ptges* knockout (**B6D:KO**) mice at the University of Connecticut Health (UConn Health). *B6D:KO* mice were then backcrossed to A/J mice for 10 generations at UConn Health (*A. 129P2(B6)-Ptges*^*tm1Dwr*^*/Drmn*, **A/D:HET**), and A/D *Ptges* knockout (**A/D:KO**) mice were generated by crossing *A/D:HET* N10 animals [6]. For co-housing experiments, two to three age-matched *A/D* and *B6D* mice were placed in a cage at 4 weeks of age and maintained for 16 weeks. Each cage housed at least 2 mice of different mouse strains and *Ptges* genotype for both males and females. For F1 hybrid mice, male *A/D* and female *B6D* mice were crossed once for each genotype, generating **B6DADF1:WT** and **B6DADF1:KO** mice. F1 hybrid mice were housed separately by the *Ptges* genotype and maintained for up to 30 weeks of age. Parental mouse lines were sacrificed at 8, 12, 16 or 20 weeks of age, and feces, whole blood, spleens, mesenteric lymph nodes (MLN) and colon tissues were collected for further analysis. *A/D:WT* and *A/D:KO* mice were littermates of *A/D:HET* breeding, and *B6D:WT* and *B6D:KO* mice were maintained by respective homozygous breeding. *A/D* and *B6D* mice were housed separately, except for co-housing experiments, and both male and female mice were used in the study. Only male mice were used for flow cytometry, lipidomics, gene expression analyses and S100a8 ELISA. Mice had access to maintenance diet (Teklad Global 19% Protein Extruded Rodent Diet) and drinking water *ad libitum*. All animal experiments were conducted with approval from the Center for Comparative Medicine (CCM) at UConn Health (AP-200208–0823).

### Flow cytometry analysis.

The spleen and mesenteric lymph node (MLN) were crushed through a 100μm nylon mesh cell strainer and washed with buffered salt solution (BSS) (n = 4 per group, male mice). Splenocytes were treated with ammonium chloride to lyse red blood cells. Immune cells were extracted according to the method by Qiu and Sheridan [19] with modifications. Briefly, colons were flushed and treated with dithioerythritol (DTE) solution to extract the IEL. After removing epithelial cells with 0.5M EDTA solution, the LP was extracted by incubating the tissues with collagenase solution and Percoll gradient. Cells were fixed with 1% formaldehyde solution and stained with CD4-PerCP and CD8-PE-Cy7 antibodies (BD Biosciences). Dead cells were excluded by staining with LIVE/DEAD Cell Imaging Kit (Thermo Fisher). FACS analyses were performed on a Becton-Dickinson LSR II (Becton-Dickinson).

### Histopathological evaluation.

For histopathologic analysis, colons were flushed with ice-cold PBS and opened flat longitudinally, fixed in 10% neutral buffered formalin solution overnight and stored in 70% ethanol thereafter. Formalin fixed colons were Swiss-rolled and embedded in paraffin for sectioning at 5μm thickness. Tissue sections were deparaffinized and stained with hematoxylin and eosin (H&E). The colon was evaluated across three distinct regions: proximal, middle, and distal. The proximal section of the colon is defined as the upper region to the point where the transverse colonic folds disappear. The rest of the colon was divided into two areas, with the proximal section defined as the middle colon and the remaining as the distal colon [20]. The number of erosions and ulcers were quantified for each section. Erosions were defined as colonic epithelium with reactive changes and/or localized dropout often associated with the infiltration of inflammatory cells into the submucosa without remodeling of the muscularis mucosa [21]. Ulcers were defined as loss of colonic epithelium and lamina propria was always associated with inflammatory cell infiltration of the submucosa with remodeling of the muscularis mucosa. Eosinophilic foci were defined as clusters of 25 or more eosinophils. Lymphoid lesions are composed of lymphoid follicles and colonic patches, the former corresponding to intramucosal collections of lymphocytes and the latter to collections of lymphocytes that reach deeper than the mucosal muscle plate. Within in the proximal region, fold enlargement was defined by accompanying inflammatory changes within the mucosa and submucosal tissue, and fold atrophy was defined as inconspicuous folds. Diameter of the lesions was measured from still images captured by Q-Capture Pro software. The percent of affected areas were calculated from the total diameter of the lesions divided by the entire length of the colon.

### RNA sequencing.

Colons were cut longitudinally, and proximal colons were snap-frozen in liquid nitrogen and stored at −80°C for RNAseq analysis (n = 12 per group, male mice). Tissues were homogenized by Polytron Homogenizer and total RNA was extracted from the tissues using RNeasy Midi Kit (Qiagen) according to the manufacturer's instructions. Quality of RNA was assessed by 2100 Bioanalyzer system (Agilent Technologies). RNA sequencing was performed at the Center for Genome Innovation (CGI), Institute for Systems Genomics, University of Connecticut as previously described with modifications [22]. Briefly, mRNA-seq libraries were prepared using the TruSeq Standard mRNA Library Prep kit (Illumina) and sequenced on the Illumina NovaSeq 600 platform. Raw reads were trimmed using Trimmomatic/0.36. The resulting reads will be mapped to the Ensembl Mus musculus genome (GRCm38.102.gtf) using star/ 2.5.3a alignment application. Reads were quantified using featureCounts (subread/2.0.3).

### Lipidomics analysis.

Colons were cut longitudinally and snap-frozen in a Precellys tube (Cayman Chemicals) in liquid nitrogen and stored at −80°C for further processing (n = 3 per group, male mice). Lipidomic analysis was performed at the Lipidomics Core at Wayne State University, as previously described [23]. Briefly, samples were homogenized and spiked with a mixture of internal standards for each individual eicosanoid (5-ng each), and biochemicals were eluted through conditioned C18 cartridges for LC-MS analysis. HPLC was performed on a Prominence XR system (Shimadzu) using a Luna C18 (3μ, 2.1×150 mm) column. HPLC eluate was directly introduced into the electrospray ionization source of a QTRAP5500 mass analyzer (SCIEX) in the negative ion mode with Multiple Reaction Monitoring (MRM). Data were collected and quantitated using Analyst 1.6.2 (SCIEX) and MultiQuant (SCIEX) software, respectively. Correction for recovery efficiencies and relative quantitation of each analyte were performed using signals in each chromatogram corresponding to the spiked-in internal standards.

### Microbiome analysis.

Fecal samples from 8- and 20-week-old mice were collected and stored at −80°C (n = 6–10 per group). Microbial DNA was extracted from fecal samples using the Shoreline Complete kit (Shoreline Biome, Farmington, CT) or Fast DNA stool mini kit (Qiagen), according to the manufacturer's protocols. For the parental mouse lines, StrainID amplicons (~ 2500 base pairs) were sequenced that encompass the entire 16S-rRNA gene, the highly variable internally transcribed spacer (ITS) region, and a portion of the 23S-rRNA gene to increase taxonomic resolution. Primers used for the amplicon synthesis had a 3-part sequence (5'-adaptor–barcode–target-specific primer-3') as described previously [24]. Briefly, the 16S adaptor sequence is 5'-GGTTATGCGGTTCACTGC-3', all barcodes were selected from the list of 384 Pacific Biosciences (PacBio)-recommended barcodes (https://www.pacb.com/products-and-services/analytical-software/multiplexing/). The target-specific forward primer sequences are a pool of primers with the sequence 5'-AGRRTTYGATYHTDGYTYAG-3'. The reverse primer had a similar 5'-adaptor–barcode–target-specific primer-3' structure, where the adaptor sequence is 5'-CGTCACTTGGCGTATTGG-3', and the target-specific sequences are a pool with the sequence 5'-AGTACYRHRARGGAANGR-3'. SMRTbell adaptors were added to pooled samples and sequencing was conducted on a PacBio Sequel 1 system. Raw sequence reads were demultiplexed using SBAnalyzer software and assigned taxonomy with strain-level resolution using the Athena Microbial Reference Database (Shoreline Biome).

Microbiome analysis of additional parental lines, co-housing and F1 hybrid mice were performed by 16S rRNA sequencing at Microbiome Research Core, University of Alabama at Birmingham.

### Short chain fatty acid (SCFA) analysis.

Fecal samples (≥ 20 mg) were collected from individual mice and stored at −80°C (n = 10 per group). Analysis was performed at the Host-Microbe Metabolomics Facility (HMMF) at The University of Chicago, as previously described [25]. Briefly, short chain fatty acids were derivatized with pentafluorobenzyl-bromide (PFBBr) and analyzed *via* gas chromatography-mass spectrometry (GC-MS, Agilent 8890) with chemical ionization (CI) with negative mode detection. The oven ramp parameters was 1 min hold at 60 ÅãC, 25 ÅãC/min up to 300 ÅãC with a 2.5 min hold at 300 ÅãC. Inlet temperature was 280 ÅãC and transfer line was 310 ÅãC. Using a HP-5ms ultra inert column (30 m × 0.25mm, 0.25 μm; Agilent Technologies 19091S-433UI), methane as the reagent gas. The instrument was in Scan acquisition mode (m/z 50–600) with a solvent delay of 4.2 with Normal Scanning and operating in negative chemical ionization mode with methane used as the reagent gas (99.999% pure). For data transformation, a 10-point calibration curve was prepared with acetate (100 mM), propionate (25 mM), and butyrate (12.5 mM) with 9 subsequent 2x serial dilutions. Data analysis was performed using MassHunter Quantitative Analysis software (version B.10, Agilent Technologies) and confirmed by comparison to retention time, nominal mass, and fragmentation patterns of authentic standards. Normalized peak areas were calculated by dividing raw peak areas of targeted analytes by raw peak areas of spiked internal standards (Cambridge Isotope Laboratories, D3-acetate DLM-3126–25; D5-propionate DLM-1601–1; D7-butyrate DLM-7616-PK; D6-phenol DLM-370-PK). Relative abundance data were normalized to the average of D7-proline/D6-phenol. Authentic standards were purchased for all targeted compounds and prepared at 1 mg/ml in methanol. All metabolite identifications are level 1 according to the CAWG standard initiative [26].

### Immunohistochemistry.

Tissue sections were deparaffinized and subjected to heat-induced antigen retrieval in sodium citrate buffer. Tissues were then incubated overnight with primary antibody for mPGES-1 (1:4000, PAB0130, Abnova) or Gr-1 (1:4000, RB6–8C5, Invitrogen), followed by an incubation with HRP-conjugated secondary antibody (Cell Signaling), and the signal was detected with DAB (Vector Laboratories) and counter-stained with hematoxylin. Images were captured using a conventional microscope using Q-capture Pro software.

### ELISA assays.

For S100a8 (Calprotectin) analysis, serum was collected in serum separator additive microcentrifuge tubes (BD) and centrifuged at 15,000 RCF for 90 seconds and stored at −80°C. Fecal samples were collected and stored at −80°C (n = 3–4 per group, male mice). Total protein from feces was extracted in a protein extraction buffer (50mM Tris, pH 7.5, 150mM NaCl) and filtered through 0.2μM column (Costar). S100a8 levels in serum and fecal samples were measured using Mouse S100A8 DuoSet ELISA (R&D Systems) according to the manufacturer's protocol.

### Statistical analysis.

Statistical analyses were performed using GraphPad Prism 10 software (GraphPad Software, Inc., La Jolla, CA) for FACS, histopathology, lipidomics, ELISA and SCFA analyses. Data are presented as the means ± SEM, and analyses were performed by one-way ANOVA with a Bonferroni comparison test. A *P*-value less than .05 (*P* < .05) was considered statistically significant. For RNAseq analyses, differentially expressed gene analysis was performed using DESeq2 R-package (version 1.42.0) with the reference level (denominator) set to *A/D:KO*. Adjusted *P*-values of the tests (adj. *P* < .05) were used to determine the significance for all analyses. GSEA were performed using gseGO and gseKEGG in ClusterProfiler, v4.9.0.002[27] with Benjamini-Hochberg corrected *P* < 0.05 as the inclusion cutoff. For microbiome analyses of parental lines, a series of custom scripts were used to survey the bacterial community compositions at the phylum, family, and species levels. Sample alpha diversity was assessed using Shannon's and Simpson's indices. Pairwise comparisons of alpha diversity were conducted using ANOVA with Tukey HSD for post-hoc testing. Differential abundance analysis was conducted at the family and species level using R package MaAsLin2[28] with “group” (A/D and B6D) as a fixed effect. For additional parental lines, co-housing and F1 hybrid mice, analysis of alpha (Shannon) and beta (Bray-Curtis) diversities were performed using R packages phyloseq as described previously [29]. Pairwise PERMANOVA and/or Kruskal-Wallis test was used to evaluate between-group differences. A *P*-value less than 0.05 (*P*<0.05) was considered statistically significant.

## Results

### A/D:KO mice develop spontaneous colonic ulcerations

**Figures 1:**
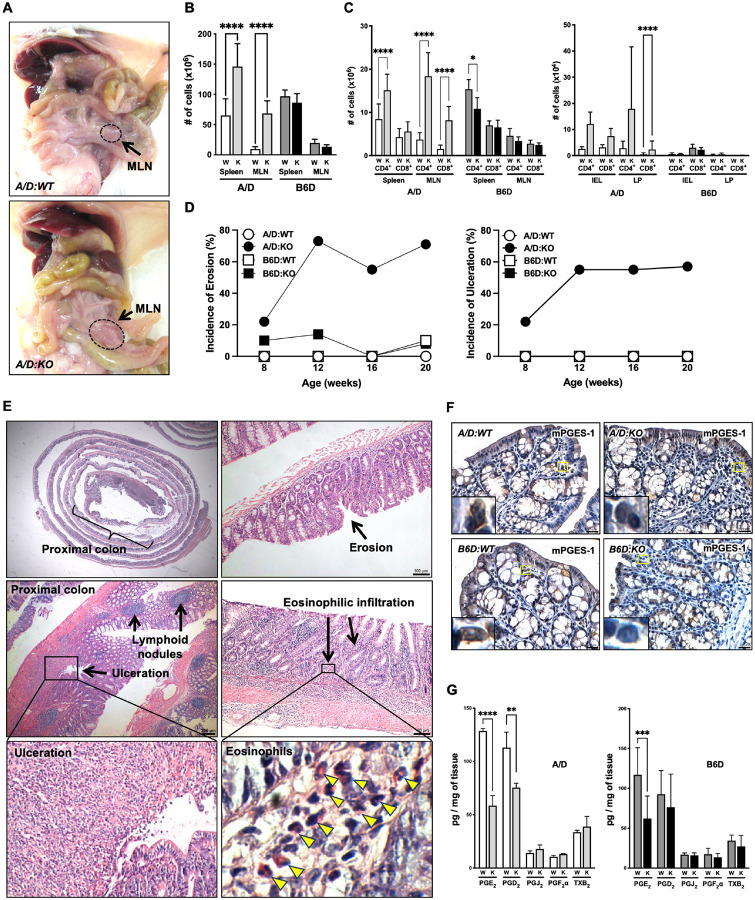
Genetic deletion of *Ptges* causes spontaneous colonic ulceration in 20-week-old mice. **(A)** Enlarged mesenteric lymph nodes (MLN) in *A/D:KO* mice. **(B)** The number of cells in the spleen and MLN. **(C)** Frequency of CD4^+^ and CD8^+^ T cells in the spleen, MLN, intraepithelial lymphocyte (IEL) and lamina propria (LP). n=10 (A/D) and n=6 (B6D). **(D)** Frequency of inflammatory lesions present in the colon at 8–20 weeks of age. n=8–14 (A/D) and n=7–12 (B6D). **(E)** Representative H&E staining of colons from 20-week-old *A/D:KO* mice, showing ulcerations, multiple lymphoid nodules, erosions and eosinophilic infiltration. The boxed area is enlarged to show an influx of eosinophils (arrowheads). **(F)** Immunohistochemistry of mPGES-1, indicating a loss of expression in the *KO* of both mouse lines. The boxed area is shown at high-power magnification. **(G)** Prostanoid levels within the colons of 12-week-old mice. W (*WT*) and K (*KO*). n=3/group. Bars indicate mean S.E.M. Groups were compared by two-way ANOVA using a Bonferroni *post-hoc* test. *adjusted *P* < 0.05, **adjusted *P* < 0.005 and ***adjusted *P* < 0.001.

We reported previously that global deficiency of *Ptges* in strain A mice (**A/D:KO**) caused localized mucosal ulcerations affecting up to 15% of the colonic epithelium, while there were no obvious phenotypic defects observed in C57BL/6 mice with the same genetic deletion (**B6D:KO**) [6, 7]. To characterize the distinct phenotype in detail, we first performed gross examination and physical evaluation from 8 to 20 weeks of age. As shown in Fig. 1A, *A/D:KO* mice had markedly enlarged mesenteric lymph nodes (MLN) compared to the *A/D:WT* mice at 20 weeks of age. Swelling of the MLN appeared as early as 8 weeks of age in the *A/D:KO* mice and was found more frequently over time (data not shown).

To assess the extent of inflammation, spleens and MLN, colonic intraepithelial lymphocytes (IEL), and lamina propria (LP) were extracted from 20-week-old mice and examined for the presence of CD4^+^ and CD8^+^ T cells. As shown in Fig. 1B, *A/D:KO* mice had significantly more T cells within the MLN compared to *WT* littermates. In addition, *A/D:KO* mice had the most significantly elevated levels of CD4^+^ and CD8^+^ cells within the MLN (Fig. 1C). In the colonic mucosa, there was a trend towards increased levels of these immune cells, with a significant increase observed in the levels of CD8^+^ cells within the LP compartment (Fig. 1C). There was no concomitant changes observed in these markers in the *B6D:KO* compared to *WT* counterparts.

To further characterize the histological expansion over time, colons were examined at 8, 12, 16 and 20 weeks of age. Colonic lesions were characterized by the extent of epithelial cell erosion and overall ulceration as described in Materials and Methods. While *B6D:KO* mice showed only minimal mucosal erosion, *A/D:KO* mice developed multiple colonic lesions at 8 weeks, progressing over time (Fig. 1D). Ulcerations were mostly confined to the proximal colon, although occasionally extending throughout the colon with associated lymphoid nodules and patches of mucosal erosion (Fig. 1E). Moreover, intraductal glandular abscesses/erosions were infiltrated with scattered eosinophils, associated with early stages of mucosal damage (Fig. 1E, **arrowheads**). However, the lesions were typically minimal-moderate, affecting less than 10% of the entire colon. This observation may explain why these mice rarely show overt symptoms of colitis, including rectal breeding and prolapse, even at 20 weeks of age.

We next examined the impact of *Ptges* deficiency on the local formation of PGE_2_ and the possibility of metabolite redirection towards other prostanoids [30]. A panel of prostanoids were measured in the colons of these mice at 10 weeks of age, prior to the appearance of detectable colonic ulcerations. Both strains of *KO* mice showed a complete loss of mPGES-1 protein expression (Fig. 1F) [6, 31], and the tissue levels of PGE_2_ were reduced to a comparable extent between these mice. There was only minimal metabolic shunting towards PGJ_2_, PGF_2_ or TXB_2_, although PGD_2_ was reduced significantly in the *A/D:KO* mice (Fig. 1G). Levels of PGD_2_ varied in *Ptges* deficient mice as increased levels of PGD_2_ have been reported in lung and macrophages [30], as well as in the stomach [32]. Functionally, PGD_2_ has been shown to have anti-inflammatory properties [33]; however, it also acts as a potent eosinophil chemoattractant during allergic reactions, thereby exhibiting pro-inflammatory properties [34]. In fact, *A/D:KO* mice show an influx of eosinophils in the colon as early as 8 weeks of age. The potential effects of PGD_2_ and eosinophils on the development of inflammatory phenotype in the *A/D:KO* warrant further investigation.

### An early-onset inflammatory response in the A/D:KO may drive the development of colonic ulceration

To identify differentially expressed genes (DEGs) associated with the divergent disease susceptibility in the two mouse lines, we performed bulk RNA sequencing (RNAseq) analysis on proximal colons of 10-week-old A/D and B6D mice, with or without *Ptges*. This is the time point immediately prior to the appearance of visible mucosal damage, in which 2 out of 9 mice developed even mild ulcerations covering less than 1% in their colonic mucosa.

**Figure 2:**
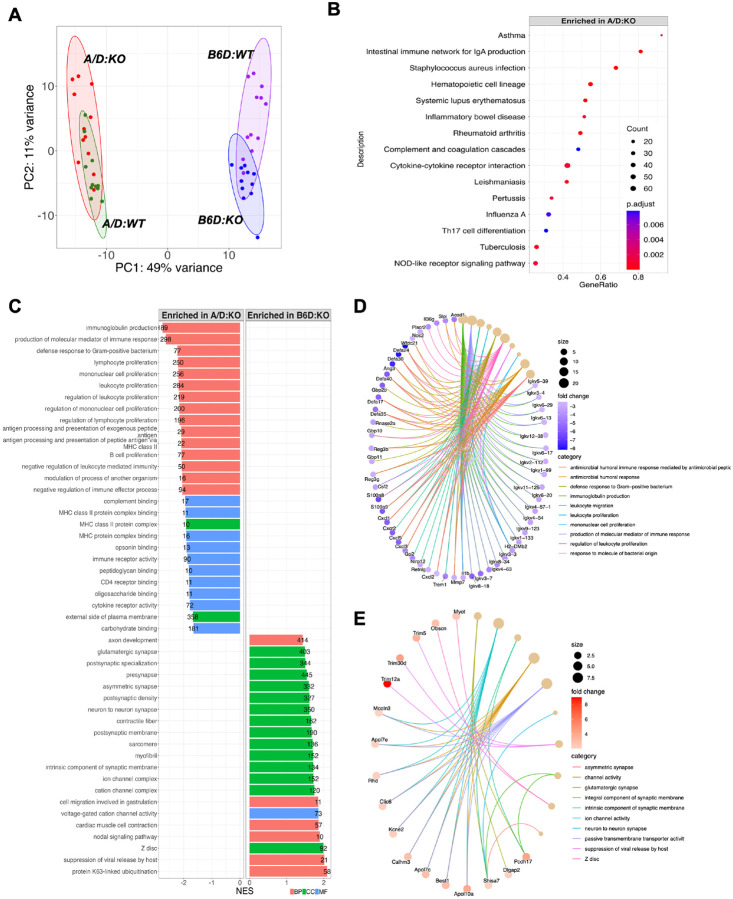
Comparison of DEGs in proximal colons between A/D and B6D mice. **(A)** PCA plot showing clear separation between the groups. **(B)** KEGG enrichment analysis. **(C)** GO analysis. **(D)** Cnet plot for *A/D:KO*-enriched DEGs. **(E)** Cnet plot for *B6D:KO*-enriched DEGs. Analyses are filtered by adjusted *P* < 0.05 and log2 FC > 1.

While intrastrain comparisons identified fewer changes between *WT* and *KO* for A/D vs. B6D (46 genes vs. 255 genes respectively; Table 1), interstrain comparisons revealed more impressive DEGs regardless of *Ptges* genotype (*A/D:WT vs. B6D:WT*, 1262 genes; *A/D:KO* vs. *B6D:KO*, 942 genes; Table 1), evident in PCA plots that showed complete separation between the two mouse lines (Fig. 2A). Despite fewer DEGs, *Ptges* status produced a wider spread of data points in the A/D compared to B6D mice (Fig. 2A), suggesting that genetic deletion of *Ptges* causes highly variable but modest levels of gene expression changes in the A/D line compared to B6D mice.

Comparing between *A/D:KO* vs. *B6D:KO* mice, KEGG pathway analysis revealed that the DEGs enriched in *A/D:KO* colons were mainly involved in inflammatory pathways, including asthma, intestinal immune network for IgA production and inflammatory bowel disease (Fig. 2B). These pathways affect various cellular processes, including mast cell activation, neutrophil/macrophage chemotaxis and modulation of antimicrobial peptides. Furthermore, Gene Ontology (GO) analysis showed that inactivation of *Ptges* in the A/D colon was predominantly associated with biological processes centered around the immune response against bacteria and/or mediated by antimicrobial peptides (Fig. 2C). In addition, these functional changes were accompanied by molecular alterations to key immune-related responses, including cytokine/chemokine activity and complement binding (Fig. 2C). *B6D:KO* colons also showed the enrichment of several functional activities related to immune response, although these pathway alterations were distinct from those present in *A/D:KO* mice. For example, pathways enhanced in the *B6D:KO* mice included suppression of viral release, regulation of lipid digestion, cholesterol absorption and the general process of post-translational modification (e.g., K63-linked ubiquitination) (Fig. 2C). Notably, several genes were associated with membrane trafficking activities involving transporters and ion channels (Fig. 2C). Cellular interactions and receptor signaling pathways support many essential functions during the inflammatory response. Thus, it is possible that some of these gene signatures represent the activation of molecular pathways in the *B6:KO* mice that contribute to the maintenance of mucosal homeostasis despite reduced PGE_2_ levels.

To gain further insight into underlying DEGs comprising these pathways, a Gene-Concept Network plot (Cnet) was generated using the top 10 GO pathways. As shown in Fig. 2D, one of the striking signatures in *A/D:KO* mice was a wide range of genes encoding the immunoglobulin (Ig) heavy and light chains, perhaps indicating the rapid activation of plasma cells associated with an adaptive immune response. This robust expression of Ig repertoires may be the basis for the extensive variability profile in PCA plot of the *A/D:KO* mice (Fig. 2A).

As shown in Fig. 2D, some of the most highly enriched genes within the *A/D:KO* colons included pro-inflammatory markers (*S100a8* and *S100a9*) and chemokines (*Cxcl2, Cxcl3, Cxcl5*), as well as various Ig genes. S100a8 and S100a9 are well-characterized pro-inflammatory proteins that are a hallmark of inflammatory diseases [35], collectively referred to as the calprotectins [36]. Fecal calprotectin levels have been shown to correlate with clinical disease activity in inflammatory bowel disease (IBD), and as such have been widely used as a biomarker for the disease [37]. Playing a crucial role in the inflammatory response, the S100a8/S100a9 complex demonstrates potent antimicrobial properties [38]. Similarly, Cxcl2, Cxcl3 and Cxcl5 are not only potent chemoattractants for neutrophil migration [39], but also display antimicrobial activity as well [40]. Indeed, the *A/D:KO* colons were also enriched with several prominent antimicrobial peptides (AMPs), including α-defensins (*Defa24, Defa30, Defa40, Defa17, Defa35*) and regenerating islet-derived protein 3-y (*Reg3y*) (Fig. 2D). α-defensins, also referred to in the mouse as the cryptdins, are proteolytically activated by matrix metalloproteinase 7 (Mmp-7) [41], which was also increased in the *A/D:KO* colons (Fig. 2D). Interestingly, Mmp-7 deficient mice show a significant decrease in PC-derived α-defensin expression, which in turn causes dysbiosis [42]. The exaggerated antibacterial response instigated by *Ptges* blockade in A/D mice suggests that their mucosal homeostasis is impaired upon their early interactions with gut bacteria.

In the colons of *B6D:KO* mice, many genes were associated with protection against tissue injury, including tripartite motif genes (*Trim12a, Trim30d* and *Trim5*) (Fig. 2E). While TRIM family of proteins regulates mucosal barrier function, they also display antiviral and antibacterial properties, directly linked to the IBD pathogenesis [43]. Moreover, expression of several Apolipoprotein L (*Apol7e, Apol7c* and *Apol10a*) genes was significantly upregulated in the *B6:KO* mice (Fig. 2E). Apol proteins serve as ion channels of intracellular membranes, which contribute to apoptosis in myeloid lineage and endothelial cells [44]. It has been reported that Apol proteins induced via Toll-like receptors (TLRs) promoted apoptosis in dendritic cells (DCs) to prevent overactivation of the immune system [45, 46].

### Fecal S100a8 correlates with the influx of neutrophils into the colons of A/D:KO mice

**Figure 3:**
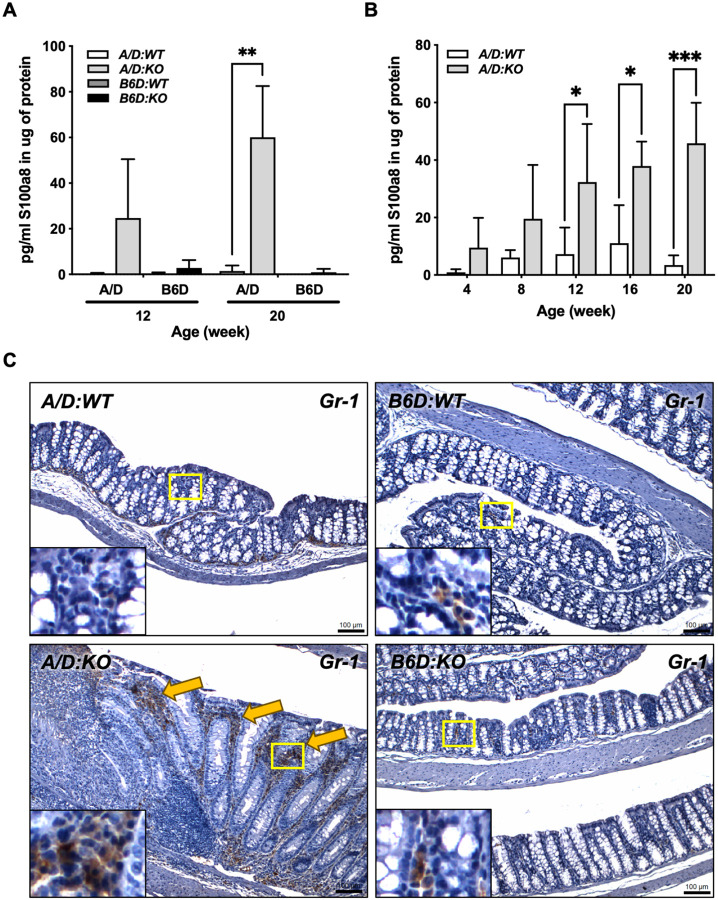
Increasing levels of fecal S100a8 and influx of neutrophils within the colons of *A/D:KO* mice. Analysis of fecal protein for S100a8 by ELISA. **(A)** Levels of fecal S100a8 in the colons at 12 and 20 weeks of age (n=3/group). **(B)** Age-dependent increase in the levels of fecal S100a8 in *A/D:KO* mice (n=4/group). Bars indicate means ± S.E.M. Groups were compared by two-way ANOVA with a Bonferroni comparison test. *adjusted *P* < 0.05, **adjusted *P* < 0.005 and ***adjusted *P* < 0.001. **(C)** Immunohistochemistry of Gr-1 in the colons of 20 week-old mice. The boxed areas are enlarged to show Gr-1^+^ cells. Arrows indicate an influx of Gr-1^+^ neutrophils within the colons in *A/D:KO* mice.

To examine potential changes in the inflammatory signature over time, we measured the levels of S100a8 in the serum and in fecal samples collected at varying times relative to the onset of colonic ulceration. While S100a8 was not detected in the serum (data not shown), *A/D:KO* mice had significantly elevated levels of fecal S100a8 at 12 and 20 weeks of age compared to the *WT* mice, whereas B6D mice of either *Ptges* genotype showed only minimal levels (Fig. 3A). Moreover, the levels of fecal S100a8 in *A/D:KO* mice increased over time (Fig. 3B), clearly correlated with disease activity (Fig. 1D). In addition, colon tissues from 20-week-old mice were examined for the presence of Gr-1^+^ neutrophils by IHC. As shown in Fig. 3C, an influx of Gr-1^+^ cells were found within the ulcerated areas of the *A/D:KO* colons, while only few positive cells were present in the colons of other groups.

These results suggest that an inflammatory response is initiated early in *A/D:KO* mice, and the resistant *B6D:KO* mice had vastly different pathway profiles from the *A/D:KO* mice, indicating that these two mouse lines activate distinct signaling systems to cope with the impaired PGE_2_ synthesis. Importantly, pathway analyses have clearly indicated the involvement of the gut microbiome in the development of colonic ulcerations observed in the A/D mice.

### Microbial community structure is affected by genetic background and Ptges genotype

Expression of genes involved in antibacterial response and barrier functions in the *A/D:KO* and *B6D:KO* colons suggest that the ensuing inflammatory phenotype may be correlated with a pathogenic microbial community structure. To explore the potential influence of the gut microbiome on the development of colonic ulcerations in the *A/D:KO* mice, we examined fecal samples collected from young adult (8-week) and mature adult (20-week) mice by 16S rRNA sequencing, as described under Materials and Methods.

**Figure 4:**
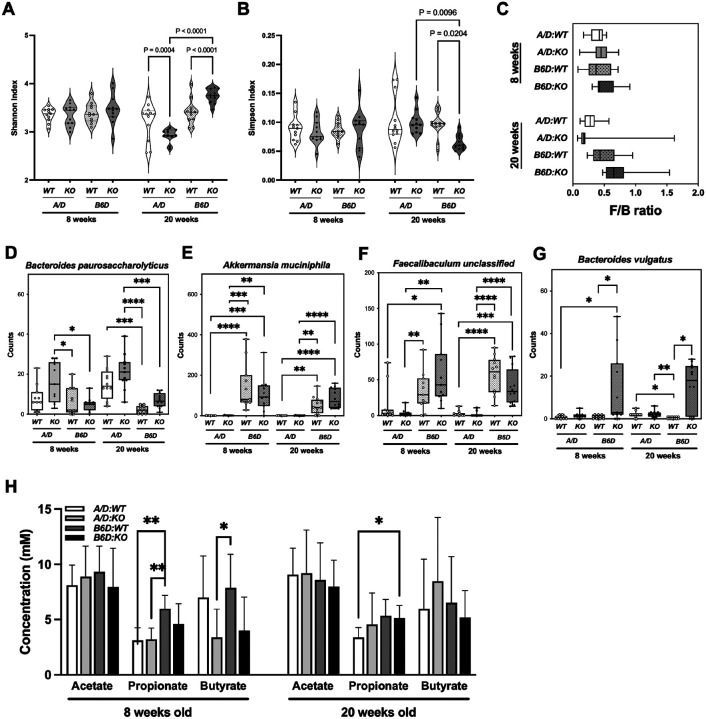
Fecal microbiome analyses in A/D and B6D mice at 8 and 20 weeks of age. Shannon **(A)** and Simpson **(B)** diversity index analyses for Species level. Violine plots show high, low, and median values. Groups were compared by ANOVA with Tukey HSD for *post-hoc* testing. **(C)**The Firmicutes/Bacteroidetes (F/B) ratio. Normalized read counts per sample for **(D)** *Bacteroides paurosaccharolyticus*, **(E)** *Akkermansia muciniphila*, **(F)** *Faecalibaculum unclassified*, **(G)** *Bacteroides vulgatus*. All *P*-values were adjusted for multiple testing using the Benjamini-Hochberg method, *adjusted *P* < 0.05, **adjusted *P* < 0.01, ***adjusted *P* < 0.001 and ****adjusted *P* < 0.0001. **(H)** Levels of acetate, propionate and butyrate in the fecal samples (n=10/group). Bars indicate means S.E.M. Groups were compared by two-way ANOVA with a Bonferroni *post-hoc* test. *adjusted *P* < 0.05, **adjusted *P* < 0.005 and ***adjusted *P* < 0.001.

As shown in Fig. 4A, alpha diversity measured by the Shannon index indicated a significant enrichment in overall microbial richness at species levels in the *B6D:KO* compared to the *A/D:KO* mice at 20 weeks (*P* < 0.0001). In addition, while *A/D:KO* mice showed a reduction, *B6D:KO* mice increased in microbial richness compared to their respective *WT* counterpart (Fig. 4A). Furthermore, the Simpson diversity index revealed that at 20 weeks, *B6D:KO* mice showed a significantly more enriched bacterial community at this level compared to *A/D:KO* mice (Fig. 4B; *P* = 0.0096). These results demonstrate that by 20 weeks, *Ptges* status, host genetic background and the presence of gut inflammation combine to exert a marked influence over the expansion/suppression of distinct bacterial species.

The ratio between the two dominant bacterial phyla (Firmicutes/Bacteroidetes, F/B ratio) are often used as a biomarker of intestinal homeostasis [47]. At 20 weeks, A/D mice exhibited a reduced F/B ratio reminiscent of IBD [47], further trending downward with *Ptges* deletion (Fig. 4C).

Several bacterial species within Phylum Bacteroidetes showed pronounced increase in the A/D mice including *Bacteroides paurosaccharolyticus* (Fig. 4D) and *Rufibacter unclassified spp*. Consistent with greater bacterial diversity (Fig. 4A), a wider array of bacterial species was present in B6D mice. For example, *Akkermansia municiphila* (Fig. 4E), was dramatically reduced within the A/D fecal stream. *Akkermansia muciniphila* degrades mucin for its source of carbon, nitrogen, and energy for survival [48], and serve as a major producer of short-chain fatty acids (SCFAs), including acetate and propionate, which in turn supports the growth of additional bacterial strains that produce butyrate [49]. SCFAs play a critical role in regulating gut homeostasis, and intestinal bacteria are the main source of these organic acids *via* degradation of dietary fibers and resistant starches [50]. In fact, several other SCFA-producing bacteria displayed distinct signatures between the four groups. For example, A/D mice harbored significantly less *Faecalibaculum spp*. (Fig. 4F), an established butyrate producer [51], which has been shown to reduce inflammation, in part by stimulating colonic regulatory T cells [51]. Furthermore, *Bacteroides vulgatus*, showed a significant expansion in the *B6D:KO* mice (Fig. 4G). Interestingly, *Bacteroides vulgatus* has been reported to attenuate the severity of DSS-induced colitis by inhibiting pro-inflammatory cytokines and modulating the growth of other SCFA-producing bacteria [52].

To determine whether these distinct bacterial signatures might be associated with the levels of SCFAs, fecal samples were analyzed by GC-MS as described in Materials and Methods. As shown in Fig. 4H, *A/D:WT* and *A/D:KO* mice had significantly lower levels of propionate at 8 weeks of age compared to *B6D:WT* mice. In addition, *Ptges*-deficient mice showed a trend towards lower butyrate levels in younger mice, an effect that disappeared as the mice aged (Fig. 4H).

Taken together, these results suggest that the A/D and B6D mice primarily harbor distinct bacterial populations, with further modifications by the absence of *Ptges*. Resultant bacterial profiles in turn are correlated with the early production of SCFAs, which may affect mucosal homeostasis later in life.

### Co-housing of A/D and B6D mice does not alter the inflammatory phenotype

Based on the distinct bacterial signatures present in these two parental mouse lines, the following cohousing study was undertaken to determine whether the microbiome present in resistant B6D mice might harbor protective bacteria that could be transferred to the sensitive A/D mice. The other possibility is that transfer of the A/D microbiome may sensitize B6D mice to mucosal damage. It has previously been shown in C57BL/6 mice obtained from different vendors that co-housing results in a nearly complete normalization of the microbiome within 4 weeks [53]. Thus, we co-housed 4-week old A/D and B6D mice of each *Ptges* genotype for a total of 16 weeks *(A/D:WT*, n = 6; *A/D:KO*, n = 10; *B6D:WT*, n = 8; *B6D:KO*, n = 8). Fecal samples were collected before (4-weeks old) and after (20-weeks old) co-housing. As shown in Table 2, fecal microbial analysis revealed a significant decrease in overall bacterial richness (*P* = 0.038, Shannon index) in *A/D:KO* mice, but a significant expansion in the abundance of microbes (Bray-Curtis dissimilarity index) in the A/D mice, with (*P* = 0.018) and without (*P* = 0.001) *Ptges*, suggesting that bacterial transfer occurred between the co-housed mice. Indeed, we identified several bacteria using Kruskal-Wallis test that were significantly decreased in *A/D:KO* mice after co-housing have been reported to exacerbate inflammatory conditions such as IBD, including *Bacteroides massiliensis* [53], Lachnoclostridium [54], and Desulfovibrio [55]. However, others have shown bidirectional effects on inflammation such as *Prevotella copri* [56]. Interestingly, there was an acquired presence of a number of bacteria in *A/D:KO* mice after co-housing, including *Akkermansia muciniphila* and *Bacteroides vulgatus* (Table 3).

Despite the successful horizontal transfer of microbiota between the two mouse lines, however, altered microbial community structure were likely insufficient to override the overall strain-related mucosal inflammation. At 20 weeks of age, 85% (11 out of 13) of the *A/D:KO* mice of either sex developed colonic ulcerations after co-housing with B6D line that mirrored the parental lines.

### F1 hybrid mice are protected from developing ulceration

**Figure 5:**
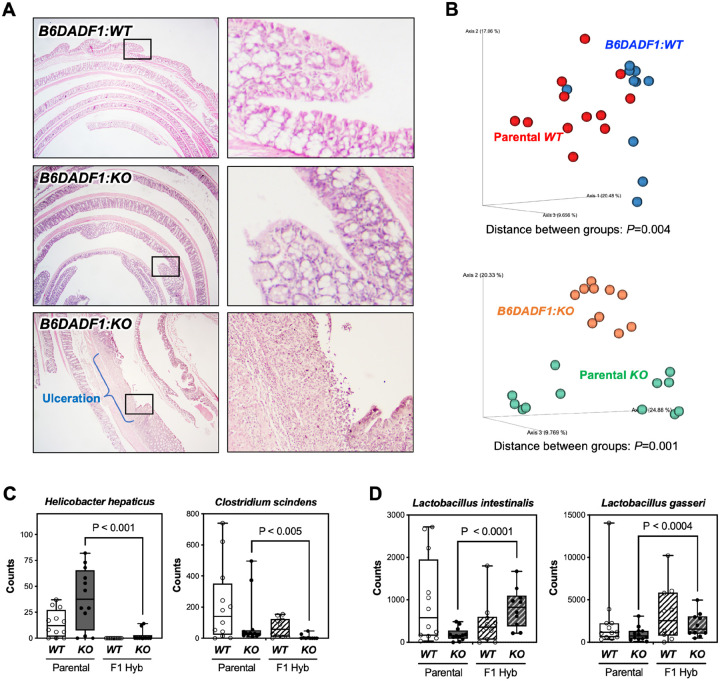
F1 hybrid of A/D:KO and B6D:KO mice do not develop colonic ulceration. **(A)** Representative H&E of colons of *B6DADF1:WT* and *B6DADF1*:*KOmice*, showing normal mucosal. Ulcerated lesion found in a *B6DADF1*:*KO* mouse. The boxed areas are shown at high-power magnification. (B) The Bray-Curtis dissimilarity index for each genotype, showing significant distance in species composition between groups. Each dot indicates individual mouse. **(C)** Read counts for *Helicobacter hepaticus* and *Clostridium scindens*. **(D)** Read counts for *Lactobacillus intestinalis* and *Lactobacillus gasseri*. Groups were compared by pairwise PERMANOVA.

We next asked whether mouse genetic background may affect the differential susceptibility to colonic ulceration. To determine the effects of gene dosage, we generated F1 hybrids by crossing A/D and B6D mice, with or without *Ptges* (*B6DADF1:WT*, n = 29 and *B6DADF1:KO*, n = 30). To standardize the effects of the gut microbiome in this study, we used female B6D mice for breeding each genotype, as the gut microbiome is primarily established through maternal transmission [57]. At 20 weeks of age, *B6DADF1:WT* and *B6DADF1:KO* mice were examined for the presence of colonic ulcerations. None of these mice exhibited enlarged spleen or MLN. Histological evaluation of the colons from *B6DADF1:WT* mice revealed no evidence of ulceration (Fig. 5A). A single *B6DADF1:WT* mouse exhibited a minute focus of eosinophils localized between the muscle layers, with a frequency likely within the range of a non-pathological condition [58]. In the *B6DADF1:KO* mice, one mouse showed a massive immune response with focal ulceration, and another presented with small lesion of much lesser extent compared to the parental *A/D:KO* mice (Fig. 5A). However, the remaining *B6DADF1:KO* mice (93%) were entirely free of colonic ulcerations, indicating nearly complete protection (Fig. 5A). To determine whether aging may affect disease onset, a subset of F1 hybrid mice (n = 30) were maintained for up to 30 weeks of age, but mice of either *Ptges* genotype remained ulcer-free (data not shown). These results indicate that the effects of genetic background have significant impact on the phenotypic variation in these mice despite impaired PGE_2_ synthesis.

To further determine the role of host-microbe interaction towards protection of colonic ulceration, we analyzed fecal samples collected from the 20-week-old F1 hybrid mice (n = 10 per group). Fecal microbiome analysis was performed by comparing between the combined parental lines (A/D and B6D) and F1 hybrids for both genotypes to identify overall changes in bacterial communities. Compared to the parental lines, microbial richness (Shannon Index) was mostly normalized at every taxonomic level in both *B6DADF1:WT* and *B6DADF1:KO*. However, the Bray-Curtis dissimilarity index indicated that bacterial community structures were significantly different between the parental lines and F1 hybrids for both genotypes (Fig. 5B; Parental *WT* vs. *B6DADF1:WT, P =* 0.004 and Parental *KO* vs. *B6DADF1:KO, P =* 0.001), suggesting that F1 hybrid lines harbor distinct microbiome. To clarify the influence of changes to microbial beta diversity on protection afforded to the colonic mucosa in the F1 hybrids, we further analyzed the data using Kruskal-Wallis test to identify bacteria that were significantly modified between the combined parental *KO* and *B6DADF1:KO* mice (Table 4). Indeed, several pro-inflammatory bacteria, including Desulfovibrionaceae [59], Roseburia [60] and *Helicobacter hepaticus* [61] were significantly reduced in the *B6DADF1:KO* mice (Table 4 & Fig. 5C). However, bacteria associated with antiinflammatory functions such as *Clostridium scindens* [62] and Oscillibacter [63] were also decreased in the *B6DADF1:KO* mice (Fig. 5C). Interestingly, the majority of the bacteria that were significantly *increased* in the *B6DADF1:KO* mice have been reported to alleviate colonic inflammation, including *Lactobacillus intestinalis* [64], *Lactobacillus gasseri* [65], Muribaculaceae [66], and Dubosiella [67] (Table 4 & Fig. 5D). In addition, a recent study has found that *Lactobacillus vaginalis*, traditionally known for its role in reducing vaginal inflammation, also ameliorates DSS-induced colitis by regulating gut microbiota balance and restoring intestinal barrier function [68]. Overall, the shift in microbial community structure in the F1 hybrid mice favors an anti-inflammatory milieu, highlighting their potential as candidates for probiotic therapy. However, a limitation of the current study is that the fecal microbiome analysis has provided only a snapshot of overall community structure present within the gut [69]. Ultimately, the potential role of altered microbial communities in the instigation of colonic ulceration should be carefully explored in future studies to capture spatially distinct niche that may be contributing to the disease onset.

## Discussion

PGE_2_ regulates discrete phases of the acute inflammatory process, including promotion of neutrophil recruitment and pro-inflammatory cytokine production as inflammation is underway [70]. Following the acute phase of tissue injury, PGE_2_ then elicits a robust immunosuppression that contributes to epithelial resolution, enabling repair and tissue regeneration [70]. Confirmation of the context-specific roles of PGE_2_ during the inflammatory response has been demonstrated in several preclinical models. For example, *Ptges-*deficient mice exhibit attenuated inflammation upon LPS challenge [18] but also display delayed recovery from tissue injury in acetic acid-treated gastric ulcers [71], as well as following dextran sulfate sodium (DSS)-induced colitis [72].

While no obvious mucosal defects have been observed with naive *Ptges KO* mice maintained on commonly used C57BL/6 [7, 18] or DBA/1LacJ [73] backgrounds, an introduction of the same genetic blockade to strain A mice causes spontaneous colonic ulceration, with mucosal damage occurring as early as 8-weeks of age. Such a marked interstrain response to the physiological stress induced by reduced PGE_2_ levels underscores the role of genetic variability in inbred mice that contributes to disease susceptibility in the colon. In earlier studies that evaluated the role of IL-10 deficiency on the development of enterocolitis [74], a widely divergent disease susceptibility was found that was entirely dependent upon genetic background [75]. For example, *IL-10* deficiency in C3H/HeJBir and 129/SvEv mice induced severe colitis, whereas BALB/c and 129xC57BL/6J mice developed an intermediate phenotype [76]. In contrast, *IL-10:KO* mice on C57BL/6J background developed only mild intestinal disease with delayed onset [76]. Interestingly, strain A mice phylogenetically related to BALB/c mice [77], and thus the shared sensitivity to induced colitis, albeit driven by different genetic stressors, may provide clues to the underlying genetics that may contribute to enhanced sensitivity.

A well-documented genetic aberration that contributes to susceptibility to inflammation in strain A mice is a loss-of-function mutation in complement component 5 (C5) [78]. Lack of C5 impairs production of pro-inflammatory cytokines from macrophages, predisposing the mice to aberrant pathogenic infections [78]. Moreover, a potentially crucial difference between strain A and B6 mice relates to their fundamental differences in the underlying biology of T helper cells. For example, whereas B6 mice generally mount a potent Th1-type response to pathogenic infections, strain A mice tend to develop a Th2-predominant response [79]. Indeed, our RNAseq data (Fig. 2B) highlight increases in a set of genes involved in complement binding and Th2-triggering events, including asthma and susceptibility to bacterial infections in the *A/D:KO* colons, gene signatures that were absent in *B6D:KO* mice. While Th2 cells contribute to repair and tissue regeneration, their excessive tissue infiltration can induce pathological fibrosis [80]. These earlier observations combined with the results of the present study suggest that A/D mice may be inherently hypersensitive to otherwise inconsequential environmental insults, generating, in turn, mucosal 'microinjuries', a direct consequence of a Th2-skewed immune system. Considering the regulatory role of PGE_2_ in controlling key aspects of inflammation, genetic disruption of *Ptges* in strain A mice may further drive subclinical inflammatory towards overt mucosal injury. In fact, a broad panel of inflammatory chemokines (Cxcls) required for neutrophil recruitment were significantly upregulated in *A/D:KO* mice prior to the appearance of discernible ulcerations (Fig. 2D), accompanied by an influx of Gr-1^+^ neutrophils surrounding the ulcerated lesions (Fig. 3C). Considering a possible threshold level of PGE_2_ that is required for neutrophil clearance, our findings suggest that continuous recruitment of neutrophils into the affected mucosa can trigger fulminant tissue damage [81]. Accumulation of these microinjuries in the tissue over time may ultimately present as more extensive mucosal damage, as evident in the *A/D:KO* mice.

Most importantly, the F1 hybrid mice (*B6DADF1:KO*), despite maintaining impaired PGE_2_ synthesis, demonstrated nearly complete resistance to colonic ulceration. This finding indicates that the parental B6D line has conferred protection to the F1 mice. On the other hand, the underlying susceptibility to colitis in the A/D line was masked in the progeny. C57BL/6 mice exhibit varying degree of resistance and susceptibility to intestinal inflammation. For example, Bouma *et al*. [82] reported that F1 hybrid mice of colitis-resistant C57BL/6 and susceptible SJL/J developed an intermediate phenotype in response to trinitrobenzene sulfonic acid (TNBS), although further resistance was present in the F2 generation. On the other hand, in the CD40-driving colitis model, F1 mice of C57BL/6 (sensitive) and BALB/c (resistant) did not show evidence of histopathologic alterations in the colon [83]. Compared to models that induce severe colitis, the extent of colonic ulceration in the *A/D:KO* mice is less severe that one or more susceptibility genes, if present, could have been inherited as recessive traits.

Alternatively, we cannot exclude the possibility that gene dosage has impacted the gut microbial community structure, in turn altering the luminal metabolome, which may have contributed to gut homeostasis in the F1 mice. It is well-established that host genetics plays an important role in shaping the gut microbiome, which, in turn, is implicated in the development of IBD-related pathologies [84]. In one such study, a combine deficiency of *Nod2* and *Cybb*, known Crohn's disease susceptibility genes, caused the accumulation of the pathobiont, *Mucispirillum scheaedleri*, that triggered the onset of colitis in a mouse model [85]. Moreover, Lamas *et al*. [86] tested another IBD susceptibility gene, *Card9*, that promotes recovery from mucosal injury by stimulating IL-22. Interestingly, *Card9-*deficient mice were found to be susceptible to colitis due to impaired tryptophan metabolism, mitigating the formation of aryl hydrocarbon receptor (AHR) ligands [86]. The administration of *Lactobacillus* strains was sufficient to reduce colitis in the *Card9:KO* mice [86], demonstrating the complex interaction between host genetics, microbial community structure and metabolite balance that collaborate to maintain intestinal homeostasis.

Whether gene-microbe interactions may play a role in *Ptges-dependent* colitis is not presently known. Indeed, the microbial composition present in the *B6DADF1:KO* were significantly different from that found in either parental line. Analysis of the community structure in the F1 hybrids identified increased abundance of several bacteria (Table 4) that are associated with protection against experimentally-induced intestinal inflammation. For example, colonization of *Lactobacillus intestinalis* has been shown to alleviate DSS-induced colitis in mice by inhibiting the production of serum amyloid A proteins (SAAs) from intestinal epithelial cells, leading to the suppression of Th17 differentiation [64]. Within the same family of bacteria, *Lactobacillus gasseri* is an established probiotic that contributes to the maintenance of healthy gut function [87].

Interestingly, several of the bacteria present in the parental *A/D:KO* feces, including *Bacteroidetes* and *Proteobacteria*, are also enriched in mice treated with various NSAIDs such as indomethacin, naproxen, and diclofenac [88]. On the other hand, Lactobacillus and Bifidobacterium families of bacteria have been tested for their probiotic efficacy to reduce NSAID-induced intestinal injuries. For example, administration of the probiotics, *Lactobacillus casei* and *Lactobacillus paracasei*, can ameliorate clinical signs of intestinal inflammation induced by indomethacin in mice [89]. Moreover, a recent double-blind, placebo-controlled trial has shown that *Bifidobacterium breve* reduces intestinal damage caused by an 8-week daily intake of low-dose acetylsalicylic acid (ASA) in healthy volunteers[90]. Based on these findings, several bacterial species found in the F1 hybrid mice, including *Lactobacillus intestinalis, Lactobacillus gasseri* and *Bifidobacterium sp*. (Table 4 & Fig. 5D) may be further evaluated as candidate probiotics for the treatment of NSAID-induced enteropathy.

## Conclusions

In summary, we have reported the development of a strain-dependent enteropathy in strain A mice that recapitulates the histologic features of NSAID-induced injury that occurs in a subset of patients on long-term drug treatment. Our results suggest that the absence of mPGES-1 activity, with associated reduced levels of PGE_2_, causes a dramatic shift in the inflammatory milieu in *A/D:KO* mice. On the other hand, the resistance to mucosal injury observed in *B6D:KO* mice underscores their ability to maintain mucosal homeostasis despite compromised PGE_2_, which may have played a dominant role in the F1 hybrid mice. Our findings warrant further studies to define the complex networks connecting inducible PGE_2_ synthesis with gene-microbe interactions. Considering the already compromised levels of PGE_2_, these genetically modified mice may serve as a useful model to study potential mechanisms to explain why a subset of patients are at greater risk of developing mucosal ulceration upon NSAID therapy.

## Figures and Tables

**Table 1:** DEGs with log2_FC > = 2 and adjusted *P*-value < 0.05.

Numerator / Denominator	Total	Numerator	Denominator
*A/D*:*WT vs. A/D:KO*	46	0	46
*B6D*:*WT vs. B6D:KO*	255	88	167
*A/D vs. B6D*	481	187	294
*A/D*:*WT vs. B6D:WT*	1262	719	543
*A/D*:*KO vs. B6D:KO*	942	525	417

**Table 2:** Alpha- and beta microbial diversity indices comparing before and after the co-housing in A/D and B6D mice.

Comparisons	n	Shannon (α)	Bray-Curtis (β)
*A/D:WT* before vs *A/D:WT* after	6	NS	↑ P = 0.018*
*A/D:KO* before vs *A/D:KO* after	9	↓ P = 0.038*	↑ P = 0.001*
*B6D:*WT before vs *B6D:WT* after	8	NS	↓ P = 0.037*
*B6D:KO* before vs *B6D:KO* after	8	NS	NS

**Table 3:** Top 40 bacteria whose frequencies were changed significantly after the co-housing in *A/D:KO* mice. c, class; o, order; f, family; g, genus; s, species.

Taxa	p-value	Kruskal-Wallis chi-squared (H)
**Decreased after co-housing**		
s_Bacteroides_massiliensis	1.33E-04	14.60
s_Alistipes_putredinis	1.33E-04	14.60
g_Acetobacter	1.33E-04	14.60
f_Ruminococcaceae	1.82E-04	14.00
s_Prevotella_copri	1.82E-04	14.00
g_Faecalibacterium	2.70E-04	13.27
g_Lachnoclostridium	3.27E-04	12.91
s_Blautia_obeum	4.83E-04	12.18
g_Subdoligranulum	4.83E-04	12.18
g_Barnesiella	4.83E-04	12.18
g_Lachnospira	4.83E-04	12.18
g_Eubacterium_xylanophilum_group	4.83E-04	12.18
s_Ruminococcus_sp.	8.11E-04	11.22
g_Agathobacter	8.11E-04	11.22
f_Ruminococcaceae	9.06E-04	11.01
g_Blautia	1.13E-03	10.61
g_Desulfovibrio	1.26E-03	10.40
g_Candidatus_Arthromitus	1.54E-03	10.03
g_Clostridia_vadinBB60_group	1.56E-03	10.01
g_Eubacterium_hallii_group	1.56E-03	10.01
**Increased after co-housing**		
s_Lactobacillus_murinus	3.49E-04	12.79
f_Desulfovibrionaceae	3.49E-04	12.79
**Decreased after co-housing**		
s_Helicobacter_ganmani	7.45E-04	11.37
g_Lactococcus	1.56E-03	10.01
s_Brachyspira_sp	1.56E-03	10.01
s_Bacteroides_oleiciplenus	1.56E-03	10.01
s_Bacteroides_sartorii	4.11E-03	8.24
s_Burkholderiales_bacterium	4.51E-03	8.06
s_Adlercreutzia_muris	1.19E-02	6.32
g_Defluviitaleaceae_UCG.011	1.19E-02	6.32
s_Bacteroides_vulgatus	1.41E-02	6.02
g_Parabacteroides	1.59E-02	5.81
o_Rhodospirillales	2.29E-02	5.18
f_Oscillospiraceae	2.91E-02	4.76
s_Alistipes_timonensis	3.05E-02	4.68
s_Parabacteroides_goldsteinii	3.11E-02	4.65
g_Gastranaerophilales	3.60E-02	4.40
g_Ruminococcus	4.03E-02	4.21
s_Akkermansia_muciniphila	4.47E-02	4.03
f_Lachnospiraceae	4.65E-02	3.96

**Table 4:** Top 40 bacteria whose frequencies were changed significantly between Parental *KO* (*A/D:KO* and *B6D:KO)* and *B6DADF1:KO* mice. c, class; o, order; f, family; g, genus; s, species.

Taxa	*p*-value	Kruskal-Wallis chi-squared (H)
**Parental > *B6DADF1:KO***		
f_Desulfovibrionaceae	1.31E-04	14.63
g_Roseburia	3.18E-04	12.96
g_Paludicola	5.85E-04	11.82
g_Alistipes	7.61E-04	11.33
g_Lactococcus	8.72E-04	11.08
g_Clostridia_UCG.014	1.16E-03	10.56
s_Mucispirillum_sp.	1.23E-03	10.44
s_Helicobacter_hepaticus	1.83E-03	9.71
c_Bacilli_RF39	1.93E-03	9.61
s_Clostridium_sp.	3.62E-03	8.47
g_Oscillibacter	4.58E-03	8.04
f_Lachnospiraceae	4.58E-03	8.04
g_Incertae_Sedis	4.58E-03	8.04
s_Clostridium_scindens	5.16E-03	7.82
g_Acetatifactor	5.62E-03	7.67
g_Erysipelatoclostridium	6.07E-03	7.53
g_Gastranaerophilales	6.55E-03	7.39
g_Tuzzerella	6.80E-03	7.33
g_Colidextribacter	7.68E-03	7.11
g_Harryflintia	9.70E-03	6.69
**Parental < B6DADF1:KO**		
s_Helicobacter_mastomyrinus	3.26E-05	17.26
s_Lactobacillus_intestinalis	1.71E-04	14.13
**Parental > *B6DADF1:KO***		
s_Lactobacillus_gasseri	3.70E-04	12.68
g_Bifidobacterium	6.06E-04	11.76
s_Lactobacillus_vaginalis	9.78E-04	10.87
g_Muribaculaceae	1.94E-03	9.60
g_Muribaculaceae	3.01E-03	8.80
o_Oscillospirales_UCG.010	3.20E-03	8.69
g_Dubosiella	3.62E-03	8.47
s_Ruminococcus_sp.	5.48E-03	7.71
g_Muribaculaceae	6.86E-03	7.31
g_Clostridia_vadinBB60_group	8.35E-03	6.96
s_Prevotella_copri	8.35E-03	6.96
g_Muribaculaceae	1.47E-02	5.95
g_Lachnospiraceae_FCS020_group	1.92E-02	5.48
s_Alistipes_onderdonkii	2.09E-02	5.34
s_Anaerotignum_lactatifermentans	2.09E-02	5.34
s_Bacteroides_massiliensis	2.48E-02	5.04
g_Muribaculaceae	2.49E-02	5.03
s_bacterium_NLAE.zl.H31	3.49E-02	4.45

## Data Availability

The authors confirm that the data supporting the findings of this study are available within the article or Supplementary Materials. GEO Accession number: GSE254848 Github link: https://github.com/micmartinezUCHC/Differential-susceptibility-to-colonic-ulceration-in-mice-with-genetic-deletion-of-Ptges

## Abbreviations

AA: Arachidonic acid
Cnet: Gene-concept network plot
CRC: Colorectal cancer
Cox: Cyclooxygenase
DSS: Dextran sulfate sodium
GI: Gastrointestinal
GO: Gene ontology
IHC: Immunohistochemistry
IBD: Inflammatory bowel disease
IL: Interleukin
IEL: Intraepithelial lymphocytes
KEGG: Kyoto encyclopedia of genes and genomes
LP: Lamina propria
MLN: Mesenteric lymph node
mPGES-1: Microsomal prostaglandin E synthase-1
NSAIDs: Non-steroidal anti-inflammatory drugs
PG: Prostaglandin
Ptges: Prostaglandin E synthase
Th: T-helper
TXB: Thromboxane
SCFA: Short chain fatty acid
